# IL-35 Regulates the Function of Immune Cells in Tumor Microenvironment

**DOI:** 10.3389/fimmu.2021.683332

**Published:** 2021-05-21

**Authors:** Kewei Liu, Ai Huang, Jun Nie, Jun Tan, Shijie Xing, Yue Qu, Ke Jiang

**Affiliations:** Department of Thoracic Surgery, Union Hospital, Tongji Medical College, Huazhong University of Science and Technology, Wuhan, China

**Keywords:** tumor microenvironment, tumor immunotherapy, regulatory immune cells, anti-tumor immunity, IL-35

## Abstract

Interleukin-35 (IL-35) is a heterodimeric cytokine composed of Epstein-Barr virus-induced gene 3 (EBI3) and IL-12p35 that has recently been shown to play diverse and important roles in the tumor microenvironment (TME). Owing to its immunosuppressive activity and ability to promote tumor growth and progression, IL-35 is widely recognized as a key mediator of TME status. Immune cells are key mediators of diverse tumor-related phenotypes, and immunosuppressive cytokines such as IL-35 can promote tumor growth and metastasis in TME. These influences should be considered together. Since tumor immunotherapy based on immune checkpoint blockade remains ineffective in many patients due to tumoral resistance, a new target or efficacy enhancing factor is urgently needed. Suppressing IL-35 production and activity has been demonstrated as an effective factor that inhibits tumor cells viability, and further investigation of this cytokine is warranted. However, the mechanistic basis for IL-35-mediated regulation of immune cells in the TME remains to be fully clarified. In the present review, we explore the roles of IL-35 in regulating immune cells within the TME. In addition, we highlight IL-35 as a specific immunological target and discuss its possible relevance in the context of immunotherapy. Lastly, we sought to summarize potential future research directions that may guide the advancement of current understanding regarding the role of this important cytokine as a regulator of oncogenesis.

## Introduction

The tumor microenvironment (TME) is a primary mediator of tumor progression, and its immunosuppressive characteristics represent a significant obstacle to solid tumor immunotherapy. The TME is composed of extracellular matrix (ECM) and non-malignant stromal cells (1). Tumor cells can readily respond to external factors and interact in a dynamic fashion with other cells within the TME (2), and the cells and cytokines within this environment can profoundly impact immune cell infiltration and proliferation and reduces the anti-cancer activity of endogenous tumor-infiltrating immune cells. Tumor-infiltrating immune cells are closely linked to tumor proliferation, angiogenesis, and metastasis (3, 4), with growth factors, chemokines, and matrix-degrading enzymes derived from these cells being conducive to aggressive tumor growth and immune responses (5–7). Immune checkpoint blockade (ICB) therapies are now used to clinically treat a range of advanced cancer types, and enhancing immune cell activation represents a novel approach to killing tumor cells (8–10). In addition, tumor cells can suppress immune responses to thereby evade detection and clearance through mechanisms including immunosuppressive regulatory cell recruitment and/or the production of inhibitory cytokines (11).

Interleukin-35 (IL-35) is an IL-12 family cytokine (12), with other members of these family consisting of heterodimeric combinations of α (p40 and EBI3) and β (p19, p28 and p35) chain subunits, including IL-12 (p35 and p40), IL-23 (p19 and p40), IL-27 (p28 and EBI3), IL-35 (p35 and EBI3), and the newly discovered member IL-39 (IL-23p19 and EBI3) (13, 14). Unlike other IL-12 family members, IL-35 has been shown to exhibit immunosuppressive activity (15). First identified by Collison et al. (16), IL-35 has since been shown to be an important regulator of tumor progression owing to its ability to drive the establishment of an immunosuppressive microenvironment (15, 17, 18). IL-35 expression within the TME can promote primary tumor cell growth and metastatic colonization at a secondary site (19, 20), signaling through a receptor composed of gp130 and IL-12Rβ2 to induce downstream Ebi3 and IL-12a transcription and the activation of a classical JAK-STAT signaling pathway (15, 21). STAT1 phosphorylation occurs upon IL-35 binding to gp130, whereas STAT4 phosphorylation occurs upon IL-35 binding to IL-12Rβ2 (22). IL-35 exert immunosuppressive action through STAT1/STAT4 in T cells and STAT1 in B cells (23, 24). IL-35 is a cytokine that is responsible for immune system maintenance and for this inhibition of immune responses, functioning by promoting the expansion of regulatory T cells (Tregs) and regulatory B cells (Bregs) while simultaneously suppressing effector T cells, Th1 cells, Th17 cells, and macrophages (25). IL-35 can control the activity of immune cells within the TME, and these cells can, in turn, regulate local IL-35 expression and function (8, 15). For example, Tregs and Bregs produce IL-35 to regulate the immune response and to facilitate tumor growth by constraining the activation of innate and adaptive immune cells (26, 27). In this review, we explore the association between IL-35 and immune cells in the TME in order to demonstrate the broad applicability of IL-35 in tumor immunology.

## The Role of IL-35 in Promoting Tumor Growth in the TME

IL-35 has been shown to play an essential role in development of benign and malignant tumors, including hepatocellular carcinoma (HCC), advanced breast cancer (BRCA), pancreatic ductal adenocarcinoma (PDAC), nonsmall cell lung cancer (NSCLC), and prostate carcinoma (PCA) (19, 20, 28, 29). Previously, Collison et al. proved that IL-35 was mainly produced by Tregs, and the expression of IL-35 in tumor cells has been gradually confirmed by Western Blot (WB) and RT-PCR analysis in recent years (19, 23). Zhu et al. found that rIL-35 can enhances malignant biological behavior of RM−1 cells in vitro compared with IL-35 neutralizing antibody treatment (19). In vivo, IL-35 has been demonstrated to promote tumor growth, progression and metastasis by enhancing the secretion of other cytokines, such as IL-6 and G-CSF (granulocyte colony stimulating factor) (30). Liu et al. confirmed that IL-35 also inhibit several cytokines including IFN-γ to achieve pro-tumor effect (31). Accumulated data indicated that IL-35 can participate in the interactions between malignant tumor cells and the surrounding immune cells in the TME, inducing an immunosuppressive environment and constraining the engagement of effective anti-tumor immune responses (32). As multiple IL-35^+^ immune cell types like M1-TAMs and DCs have been discovered and isolated, current evidence indicates that tumor-derived IL-35 has been widely implicated in pro-tumorigenic properties of different cellular contexts, likely *via* suppressing tumor-infiltrating lymphocytes (TILs) infiltration and effector cell proliferation (20, 32, 33). Overall, IL-35 produced by the malignant tumor cells as well as surrounding stromal cells contributes to immunosuppression within the tumor microenvironment thereby supports sustained tumor growth and metastasis.

## IL-35 and Immune Cells in the TME

TME consist of functional altered stromal cells [cancer-associated fibroblasts (CAFs), vascular endothelial cell (VEC)], myeloid populations [dendritic cells, macrophages, and myeloid-derived suppressor cells (MDSCs)], and TILs [T cells, B cells, monocytes, tumor-associated neutrophils (TANs) and natural killer (NK) cells)]. In early 2016, Pylayeva-Gupta et al. have mentioned that one of the IL-35 receptors, gp130, has been found on multiple immune cell types and the potential role of IL-35 was conceived through association involving tumor and stromal cells (34). In addition to the widespread expression of IL-35, immune cells are the main producers of IL-35 compared with tumor cells. While previous articles regarded Tregs or iTr35 cells as significant IL-35 producers (Collison et al.), IL-35 is also secreted by B cells, DCs, endothelial cells and macrophages (16, 35). In addition, NK cells, Tans, monocytes and MDSCs can also participate in bidirectional interactions between IL-35 and immune cells above (36). At the same time, IL-35 is involved in the myeloid cells recruitment and suppress differentiation of anti-tumor cytotoxic T lymphocytes (CTLs), NK cells and other effector immune cells at the tumor site (36, 37).

### Interactions Between IL-35 and T Cells in the TME

**Figure 1:**
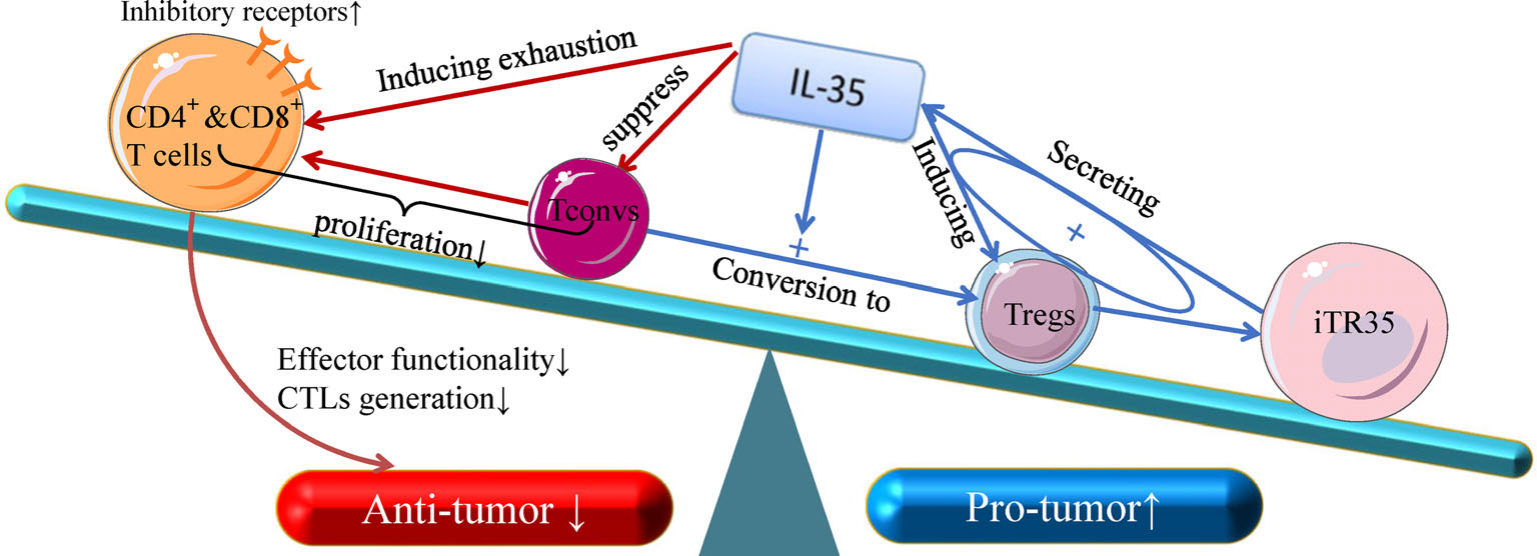
The anti- and pro-tumor effects of T-cell subsets in response to IL-35. Recent insight into the biology of IL-35 suggests that it can both suppress naive T cells (Tconv cells) and convert these naive Tconv cells into strongly suppressive induced Treg cells (iTr35 cells). Through a positive feedback loop associated with the expression of IL-35, Treg cells can inhibit the differentiation of CD4^+^ T cells into Th17 and Th1 cells. In addition, IL-35 is dispensable for the control of CD8^+^ T cells, and IL-3- dependent transcription signal depletion as well as the inhibitory receptor activation of CD8^+^ T cells play key roles in reducing CTL effector functions, which are critical for cellular anti-tumor responses. Given its broad immunoregulatory properties and its pro-tumor functions, IL-35 represents an important functional cytokine for Tregs that is secreted by multiple immunosuppressive cell subsets.

T cell interactions with tumor cells in the TME has the potential to result in T cell activation and spontaneous anti-tumor immunity (38). IL-35 can influence the transcription and expression of T cell immune response-related differentially expressed genes in tumor tissues (31), but the immunosuppressive activity of this cytokine is primarily attributable to its inhibition of CD4^+^ and CD8^+^T cells and of anti-tumor immune responses (**Figure 1**) (39). IL-35 derived from Tregs within tumors can induce CD4^+^and CD8^+^ T cell exhaustion, reducing their effector functionality and impairing the generation of CTLs (31, 40). However, IL-35 does not directly suppress these CTLs, instead downregulating the costimulatory molecule CD28 on the surface of immature CD8^+^ T cells and thereby interfering with their ability to differentiate into anti-tumor CTLs. IL-35 can also disrupt Th1 cell activation to suppress differentiated anti-tumor CTL activity (41). IL-35 does not alter the survival of the A549 ADC cell line or the H520 SCC cell line cultured with IL-35, suggesting that the immunosuppressive functions of IL-35 necessitate the presence of the TME *in vivo*, as this cytokine does not seem to influence tumor cells *in vitro* (39). Together, these results suggest that IL-35 can suppress the activation of tumor-infiltrating T cells and anti-tumor CTLs, specifically promoting tumor development within the TME (31, 39, 41).

Tregs are key immunomodulatory cells within the TME, wherein the control tumor growth, migration, and local immunity (42, 43). Tregs have been shown to secrete both IL-10 and IL-35 (16, 44), and are present within tumor-draining lymph nodes wherein they can be stratified into Treg subsets secreting either IL-10 or IL-35. Cooperation between these two cytokines can drive conventional T cell failure and BLIMP1 upregulation-mediated transcriptional signal depletion in tumor-infiltrating CD8^+^T cell (45, 46). IL-35 can influence the function of T cells by modulating their expression of inhibitory cell surface receptors (IRs) and their ability to secrete effective cytokines (31). Inhibitory receptors including CTLA4 (cytotoxic T lymphocyte-associated protein 4), PD-1 (programmed cell death ligand 1 or CD279), Tim-3 (T-cell immunoglobulin and mucin-domain containing 3), and LAG-3 (lymphocyte activation 3) have been identified as hallmarks of dysfunctional T cells (47). IL-35 can specifically increase the expression of these receptors on CD4 and CD8^+^ tumor-infiltrating lymphocytes (TILs) within the TME, thereby depleting local T cells (45, 46). Tregs can also convert between the expression of IL-10 and IL-35, enabling them to adapt to the dynamic TME (40). Conventional T cells can differentiate into Tregs in response to IL-35-mediated STAT1/STAT3 signaling, yielding so-called iTr35 cells (48), which can mediate the relationship between IL-35 and tumor cells in TME, accelerating tumor cell growth and metastasis (22, 28). In addition, iTR35-derived IL-35 can drive the differentiation of other Tregs towards an iTr35 phenotype, creating a positive feedback loop that can control TME activity over extended periods of time (48, 49). Notch signaling blockade can also alleviate the inhibitory functions of purified Tregs from gastric carcinoma patients, which can increase the secretion of IL-35, confirming the close feedback relationship between IL-35 and Tregs (50).

Intriguingly, IL-35 improve anti-tumor activity *via* the WNT/β-catenin pathway, suggesting that IL-35 plays distinct roles in different signaling pathways (51). The activation of the β-catenin pathway has recently been shown to be associated with most human tumors exhibiting spontaneous T cell infiltration (52). β-catenin maintains Treg function and contributes to regulated IL-10 production within the TME, and its expression is inhibited by IL-35 (53–55). Overexpressing IL-35 inhibits colon cancer cell migration, invasion, proliferation, and colony formation by suppressing this β-catenin pathway (51). The WNT/β-catenin pathway may thus represent a novel mechanism whereby IL-35 can influence tumor progression, highlighting this as an additional avenue for the study of IL-35 use in the context of tumor immunotherapy.

### Interactions Between IL-35 and B Cells in the TME

The role of B cells within the TME remains a matter of controversy, with some evidence suggesting that these cells can promote or inhibit tumor progression *via* a range of immunomodulatory mechanisms (8, 56). B cells are known to regulate immunity by producing specific antibodies, forming antibody-antigen complexes, secreting cytokines, and serving as antigen-presenting cells to promote cytotoxic T cell responses (57, 58). Tumor-infiltrating B cells in the TME have been shown to promote tumor progression through interactions with macrophages, MDSCs, and other immune cells by secreting cytokines such as IL-10, TGF-β, and IL-35 (56, 59). B cells also exhibit distinct phenotypes under different TME conditions, with Bregs being a recently identified B cell subtype exhibiting immunosuppressive functions (60, 61). Much like Tregs, Breg recruitment can result in the modulation of local cellular responses through the production of IL-35 and other regulatory cytokines (62). In mouse models of breast cancer, Bregs induce T cell differentiation into Tregs and thereby accelerate tumor growth and lung metastasis (63).

**Figure 2:**
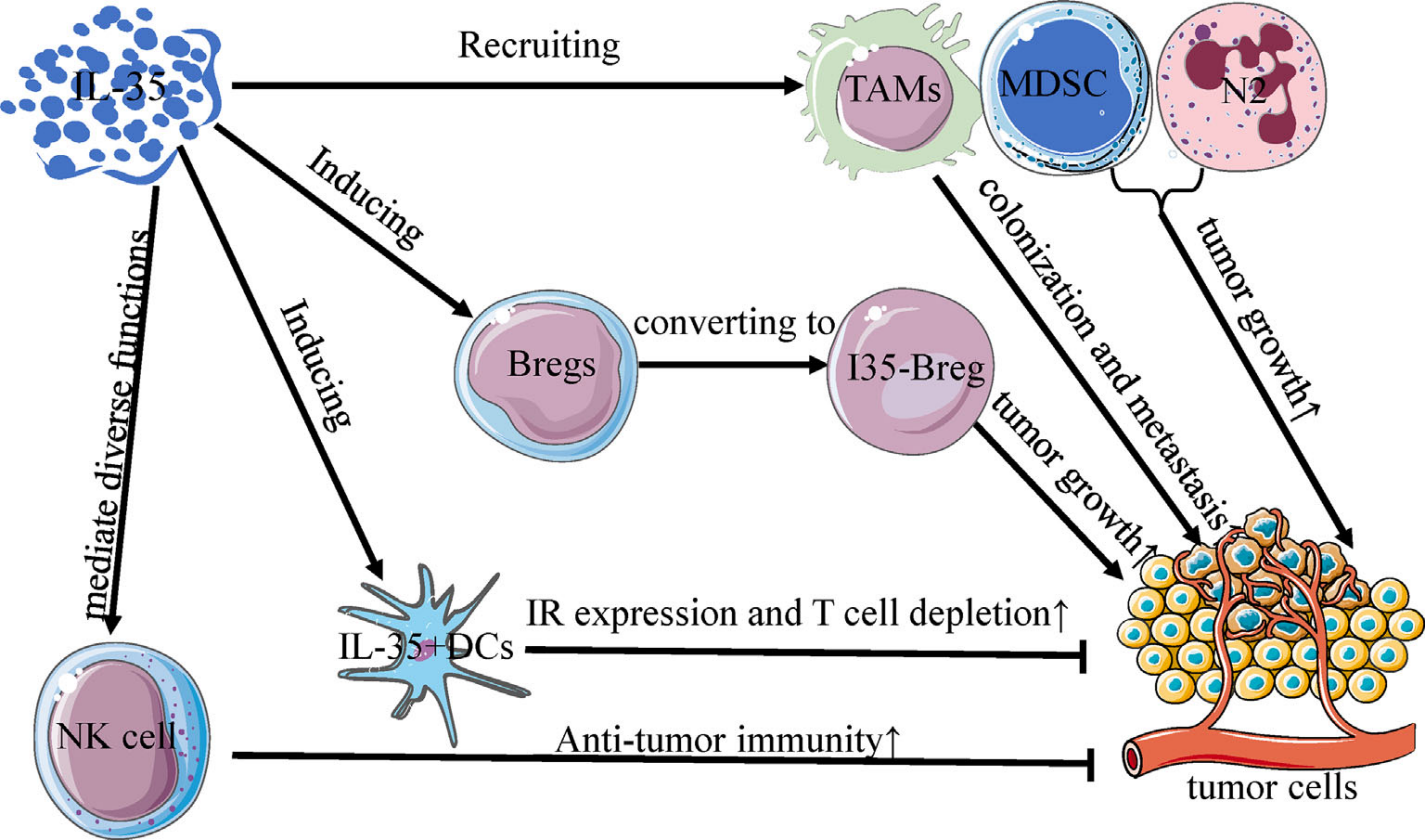
Interactions between IL-35 and other immune cells in TME. IL-35 regulates the activity of immune cells in the tumor microenvironment, and is mainly produced by Bregs, DCs, NK cells, TAMs, MDSC, and N2 neutrophils. Much like the differentiation of Treg cells, IL-35 can convert Bregs to an active subset (I35-Bregs) which secrete IL-35 to promote tumor growth. With increased inhibitory receptor expression and T cell depletion, IL-35^+^ DCs can slow primary tumor growth rates, unlike other IL-35^+^ immune cells. Furthermore, IL-35 mediates diverse functions in NK cells and is obligatory for promoting the early NK cell-mediated responses to enhance primary anti-tumor immunity. TAMs, MDSCs, and N2 cells recruited by IL-35 can similarly potentiate primary tumor growth and metastatic colonization in the tumor microenvironment.

Several early studies of Breg-like cells that produce IL-10 (B10 cells) were conducted, underscoring the regulatory roles of these cells in a range of different tissues and immunological contexts (64, 65). However, recent evidence suggests that IL-35 is a key Breg cytokine that can drive conventional B cells and B10 cells towards an IL-35-producing B cell (i35-Breg) phenotype (57, 66). In the TME associated with gastric and pancreatic tumors, these i35-Bregs are significantly more abundant and can promote tumor progression (**Figure 2**) (37, 67). In pancreatic tumors, inhibition of the Bruton’s tyrosine kinase (BTK) signaling pathway reduces IL-35 and IL-10 expression and decreases i35-Breg cell function, suppressing tumor cell growth (67). Wang et al. determined that numbers of i35-Bregs were significantly increased in those with advanced gastric cancer, and found that the amount of these cells was associated with quantities of Tregs, MDSCs, B10 cells, and CD14^+^ monocytes in the patients (37). As a specific B cell subtype, i35-Bregs can regulate other stromal cells within the TME including effector T cells, tumor-infiltrating MDSCs, NK cells, and macrophages (35, 37). As the specific molecular markers of Bregs are unclear, however, future in-depth analyses of IL-35 and Bregs are required to understand the immunological effects of i35-Bregs (37, 62).

### Interactions Between IL-35 and Dendritic Cells in the TME

Dendritic cells (DCs) are key professional antigen-presenting cells (APCs) that promote antitumor immune responses by regulating T cell activation and proliferation, modulating immunological homeostasis within the TME. The migration of activated DCs into lymphatics can efficiently present antigens to T cells (68). DCs play a critical role in the initial activation of antitumor immunity by serving as an essential interface between antigen-independent innate immunity and antigen-specific adaptive immunity (69). Restoring the antigen-presenting activity of DCs thus represents an important approach to achieving efficacious tumor immunotherapy outcomes (69, 70). Seyerl et al. were the first to show that human rhinovirus-activated DCs (R-DCs) produced IL-35, driving CD4^+^ T cell development into ITR35 cells (33). DCs have also been shown to markedly upregulate IL-35 in response to lipopolysaccharide (LPS) stimulation, and there is evidence that IL-35 can suppress such LPS-triggered DC maturation and can influence the ability of DCs to produce factors including IL-10 and TGF-β (71, 72). IL-35^+^ DCs and interacting T cells enrich P35 and Ebi3 expression, and mice vaccinated using IL-35^+^ DCs exhibit enhanced tumor growth and reductions in T cells within the TME, consistent with the ability of this cytokine to constrain intratumoral immune responses (73, 74). Cytokine-induced killer (CIK) cells, a type of MHC-unrestricted CD3^+^CD56^+^ cytotoxic T lymphocytes, have also been found to exhibit anti-tumor activity as an immunotherapy target in certain malignancies (75, 76). Tregs and IL-35 levels increase in a time-dependent fashion in the context of CIK cell production, whereas DCs can inhibit such increases and enhance CIK cell cytotoxicity (77). As dendritic cell (DC)-CIK-mediated immunotherapy is an effective method for adoptive cell therapy, the potential role of IL-35 in combined CIK/DC-CIK therapy and chemotherapy warrants additional consideration (78, 79). IL-35 can also convert immunogenic CD8α-DCs into tolerogenic DCs. Such tolerance-induced DCs (TolDCs) also express IL-12p35 and Ebi3, and can upregulate Ebi3 and IL-12p35 when stimulated with IFN-γ, LPS, or CD40L (71). Such TolDCs may thus represent an important focus for future studies of interactions between IL-35 and DCs. Arginase-1 (Arg1) is a metabolic enzyme specifically expressed by DCs that functions as an immune checkpoint molecule associated with tumor immune escape, and it is also a downstream effector of IL-35 (39, 77). Downregulation of IL-35 within the TME can inhibit Arg1 expression and immunomodulatory activity, inducing immune escape and tumorigenesis (77).

### Interactions Between IL-35 and Macrophages in the TME

Macrophages are among to most abundant immune cells within the TME, and they are broadly classified into the inflammatory, classically-activated M1 subtype and the pro-tumorigenic, alternatively-activated M2 subtype, which are referred to as tumor-associated macrophages (TAMs). TAMs, together with DCs, are members of APC populations in the TME and play important roles in tumor growth and progression, depending on their relative M1 or M2 polarization, respectively (80, 81). Relative to metastatic tumors, M2 markers are less abundant within primary tumors, wherein macrophages exhibit distinct activity. EBI3 and IL-12a, two subunits of IL-35, have been shown to be highly expressed in metastatic tumor-associated TAMs together. M2 cells in both humans and animal models express the IL-35 EBI3 subunit, which promotes macrophage differentiation, survival, and recruitment into the TME and maintains M2-like TAM cell functionality (20, 39, 82). Recruited by IL-35 from PDAC cells, macrophage express CXCL1 and CXCL8 to promote angiogenesis (83). Deletion of EBI3(EBI3^L/L-Tom^) induced a decrease of M1-TAM population, while Sawant et al. observed a comparable advancement of it in IL-10-deficient mice (IL-10^L/L^) (46). The biological interaction between IL-35 and M2 is also affected by some factors. IL-35 of M2 macrophages was suppressed by NRP1 expression in Stomach Adenocarcinoma (STAD), and the expressed cytokine was served as a major signal in the immune suppression mechanism of STAD (84). In the context of NSCLC, Heim et al. found that increased Arg1 mRNA expression was positively correlated with levels of IL-35^+^ M2 cells and Tregs, whereas TNF-α expression fell as IL-35 expression increased. This suggests that IL-35^+^ M2 cells produce Arg1 mRNA to induce or attract the production of iTr35 cells, thereby driving IL-35 production and associated responses (39). The number of CD68^+^ macrophages in tumors has also been shown to decline and to be positively correlated with IL-35 levels in normal tissue, suggesting that there may be a distinct subset of macrophages expressing IL-35 within the TME (39). TAMs within primary tumors mainly secrete TNF-α to induce the process of epithelial-mesenchymal transition (EMT) which is an indispensable process that enhances the migratory and invasive abilities of tumor cells (20, 85). At metastatic sites, in contrast, TAMs produce IL-35 to reverse the EMT by activating JAK2-STAT6, GATA3, and other signaling pathways, thereby facilitating metastatic tissue colonization (20). Such TAM-mediated activity is believed to be a primary driver of NSCLC metastatic colonization of distal tissue sites (20).

### Interactions Between IL-35 and Endothelial Cells/Monocytes in the TME

Angiogenesis, this process of tumor vascular growth is an inevitable program of tumor progression in the TME. It can promote the proliferation of epithelial cells by releasing several immunosuppressive cytokines and angiogenic growth factors. While secreted by endothelial cells and monocytes to less extent, the key role of IL-35 in angiogenesis should not be ignored. Wang et al. established a J558 mouse model and determined that IL-35 can induce the expression of CD31 and vascular endothelial growth factor (VEGF), inducing angiogenesis and endothelial cell activation (86).IL-35 also facilitates PDAC endothelial adhesion and transendothelial migration *in vitro via* the endothelial adhesion molecule, ICAM1 (29). With establishing a model system similar to the TME, Liao et al. confirmed that IL-35 from tumor cells, Tregs, and MDSCs promote the secretion of VEGF to attracts endothelial cells. In addition, monocytes are central players in angiogenesis and the source of pro-angiogenic cytokines. In early 2018, Huang et al. discovered that IL-35 increased the recruitment of monocytes to promote PDAC progression and monocyte-induced angiogenesis through IL-35-CXCL5 axis (C-C motif chemokine ligand 5) by RNA-seq and immunohistochemical analyses in xenograft mouse models (83). In PDAC samples from patients, they measured markers that can reflect the expression level of IL-35, such mRNA and protein. Not unexpectedly, the markers correlated with microvessel density and infiltration of monocyte lineage cells (83). Subsequently, Wang et al. identified that numbers of i35-Bregs were remarkably increased with the accumulation of CD14^+^ monocytes (37). Moreover, IL-35 suppressed CD14^+^ monocytes induced naive CD4^+^ T cell activation and the production of TNF-α and granzyme B, inhibiting cytotoxicity of them, whereas this effect has not been reported in TME (87). Further studies are needed to elucidate that function of IL-35 in the complex role of endothelial cells and monocytes during the angiogenesis in the TME.

### Interactions Between IL-35 and Other Immune Cells in the TME

Prior studies have explored the interactions between IL-35 and other intratumoral immune cell populations not discussed above, such as neutrophils, MDSCs, and NK cells (88–90), although more research on these interactions is warranted. Anti-tumorigenic N1 neutrophils and pro-tumorigenic N2 neutrophils are two different subsets of neutrophils identified in previous studies (91). IL-35 has been shown to promote N2 neutrophil polarization by increasing G-CSF and IL-6 production, thereby enhancing the ability of these N2 cells to promote angiogenesis and to suppress immune responses, thereby enabling invasion of N2 cells into tumor tissue (30). MDSCs within the TME can secrete a range of immunosuppressive factors including IL-35, and in PCA model mice, high IL-35 levels were associated with increases in MDSC and Treg levels and reductions in CD4^+^ and CD8^+^ T cell frequencies within the spleen, blood, and TME, and with alterations in tumor growth, metastasis, and worse survival outcomes (19). Moreover, tumor-cell derived IL-35 was reported to promote tumor growth and angiogenesis through the enhancement of myeloid cell accumulation in the TME (92, 93). NK cells are important mediators of anti-tumor immune responses, and cytokines including IL-35, IL-12, IL-23, and IL-27 can modulate DC, macrophage, and NK cell functionality to directly and indirectly control NK cell immune responses (36, 94). While the mechanistic basis for these interactions remains to be clarified, there is clear evidence that IL-35 is likely to regulate a range of immune cell functions within the TME.

## Exploration of IL-35 and PD-1/PD-L1 in Cancer Immunotherapy

Programmed cell death protein 1 or its ligand (PD-1/PD-L1) are both important regulators of the TME (95, 96), and the emergence of antibody-based ICB therapies against PD-1/PD-L1 has led to an improved immunotherapy method of several cancer types (97). However, these treatments are effective only in a minority of cancer patients. In the present study, scholars sought to address those issues, and the interaction between IL-35 and PD-1/PD-L1 discovered by animal models or clinical samples may be a potential solution of it. Through Treg-restricted deletion of Ebi3 (subunit of IL-35), Turnis et al. originally demonstrated that IL-35 in tumor-infiltrating Treg cells can promote expression of PD-1 on the surface of the B16 tumor model T cells, leading to exhaustion of them (32). Sawant et al. similarly produced a loss of Treg cell-derived IL-10 or IL-35 model mice and observed a comparable reduction of PD-L1 expression to confirm that these cytokines can drive coordinated conventional T cell failure up regulating expression of PD-1. Yang et al. proved that IL-35 also stimulates PD-1 in peripheral CD8^+^T cells in addition to tumor-infiltrating CD8^+^T cells in HCC (98), and Dong et al. pointed-out that PD-L1 promotes the expansion of regulatory T cells and recruit more IL-35 in the TME, leading to AML cell proliferation (99). The connection between IL-35 and PD-1/PD-L1^+^ non-T cells was also found beyond PD-1/PD-L1^+^ T cells. Takahashi noticed that depletion of IL-35 decreased the number of PD-L1^+^ B cells and constrains pancreatic tumor cell growth in the pancreata of Kc-IL-1β mice, an artificially constructed hybrid mouse overexpressing IL-1β (100).

**Figure 3:**
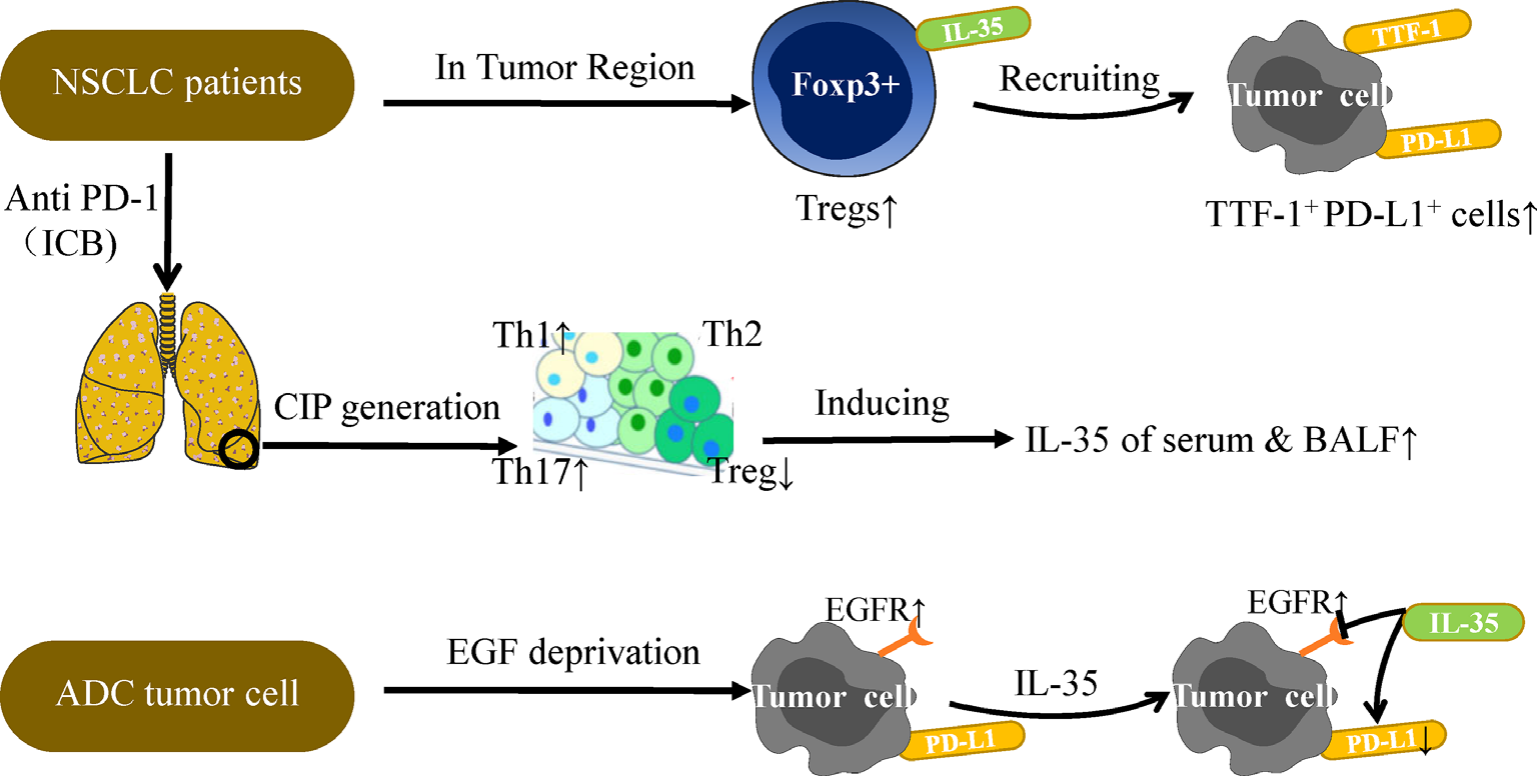
IL-35 and PD-L1 in NSCLC immunotherapy. PD-L1 expression on tumor cells is the most studied biomarker in the context of predicting NSCLC patient immunotherapy outcomes. Further studies are needed to explore the relationship between IL-35 and PD-L1. Under conditions of EGF deprivation, IL-35 can reduce the expression of PD-L1 without impacting EGFR, which is also a target of NSCLC immunotherapy. A positive correlation between IL-35^+^Tregs and TTF-1^+^PD-L1^+^ cells has been observed in the TU region, and TTF-1 is a good prognostic factor associated with survival in NSCLC. In addition, CIP generation is often observed in patients undergoing PD-1 or PD-L1 antibody therapy. CIP patients have higher mean amounts of Th1 and Th17 cells and lower levels of Tregs in serum as compared to normal subjects, thus inducing IL-35 secretion in the plasma and BALF. The levels of IL-35 in the plasma and BALF can be measured for the diagnostic evaluation of CIP patients. Overall, the complex relationship between IL-35 and PD-L1 must be considered when discussing the potential role of IL-35 in tumor immunotherapy.

Subsequently, the clinical samples confirm the above findings again. In NSCLC patient tumor tissues, IL-35^+^ Tregs have been found to be positively correlated with TTF-1^+^ (Thyroid transcription factor) PD-L1^+^ cells confirmed by immunohistochemistry (IHC) staining (39). However, under conditions of growth factor-deprivation that yield cells sensitive to EGF (epidermal growth factor), IL-35 can inhibit expression of PD-L1^+^ in the ADC (lung adenocarcinoma) tumor cell line without impacting EGFR (epidermal growth factor receptor) (**Figure 3**) (39). The above researchers have verified, extended and summarized the interaction between IL-35 and PD-1/PD-L1 on the surface of immune cells. However, in the DLBCL cases analyzed by Larousserie et al, a positive correlation between expression of IL-35 and PD-1 was not observed (101). This suggests that the administration of an anti-IL-35 antibody may be of value in the context of anti-PD-1 treatment, but it still needs to be further studied (39). Anti-PD-1 treatment can drive the activation of TILs that have otherwise lost their immunoreactive functionality, thereby restoring their proliferation and cytotoxicity such that the prognosis of treated treatment of NSCLC patients can be significantly improved (102, 103). Checkpoint inhibitor pneumonia (CIP) is a particularly dangerous form of immune-related adverse events (IrAE) that can arise in NSCLC patients receiving anti-PD-1/PD-L1 therapy (104, 105). Wang et al. discovered that the occurrence of CIP changes the proportion of T cell subsets in plasma, thereby promoting increased secretion of IL-35 in plasma and BALF (106). As such, measuring IL-35 levels may offer insight into the risk of developing CIP, and IL-35 may also represent a viable therapeutic target in patients undergoing tumor immunotherapy.

## Conclusion

Undoubtedly, IL-35 is a regulator of tumor progression via the control of immune cell activities within the TME. IL-35 is primarily secreted by iTR35, and also secreted by tumor cells and other non-T immune cell subsets. IL-35 expression is evident on the surfaces of many immune cell types, potentially further influencing its ability to impact oncogenesis. Previous studies have revealed that tumors escape immune surveillance and immune attack to achieve tumor progression and metastasis by IL-35 in conjunction with regulatory immune cells (86). As discussed above, IL-35 is thought to be an important mediator of the host immune response and tumor survival which has specific immunosuppressive activity, functioning by modulating local inflammatory responses and anti-tumor immunity in the TME (32). The discovery that IL-35 can induce the angiogenesis by monocyte/macrophage which secrete this cytokine offers key insights that can guide future studies of the role of IL-35 in cancer and immunotherapeutic treatment. The expression and promoting effect of IL-35 in tumor have been described in plenty of malignant tumors, such as HCC, PDAC & PCA (19, 20, 29). As a result of the “cold” nature of these tumors, patients receive little benefit from conventional immunotherapy treatments, and by developing a more comprehensive understanding of the mechanisms governing the production and signaling activities of IL-35 it may be possible to develop better targeted anti-tumor treatment strategies for these patients. Controlling the expression of IL-35 so as to prevent it from driving tumor proliferation and metastasis thus represents a promising direction for immunotherapeutic efforts.

While we herein systematically discussed important findings pertaining to regulation of the TME or immune cells by IL-35 that have been published over the past decade, these two IL-35-dependent activities are inextricably linked to one another but the degree to which these activities influence tumor progression in different tumor types remains to be clarified. In addition, the specific roles of various immune cell types in the context of IL-35-mediated interactions within the TME remain to be fully elucidated. Studies regarding the immunosuppressive functions of IL-35 are also at a very early stage, and further preclinical research is warranted. Future studies should place more emphasis on the development of IL-35 as a therapeutic target to interfere with tumor growth and to assess the physiological impact of IL-35 on the primary tumors in human clinical trials. In this review, we systematically discussed the recent advances in our understanding of IL-35 as a regulator of immune cell regulation and proliferation, thereby highlighting a potential target for tumor immunotherapy. Given the novel role of IL-35 in creating an immunosuppressive tumor microenvironment, the tumor-related mechanisms whereby this cytokine and other IL-12 family members function warrant additional detailed research, as they may generate novel immunotherapeutic approaches and give rise to exciting clinical applications.

## Author Contributions

KL and AH contributed equally to this review. KJ conceptualized the review and finalized the manuscript preparation. KL and AH performed the literature search and drafted the manuscript. JN, JT, SX, and YQ modified the grammar of this review. All authors contributed to the article and approved the submitted version.

## Funding

This work was supported by the National Natural Science Foundation of China (No. 81974038), the Hubei Provincial Natural Science Foundation of China (No. 2017CFB615).

## Conflict of Interest

The authors declare that the research was conducted in the absence of any commercial or financial relationships that could be construed as a potential conflict of interest.
